# Suspicious looking mu rhythm on SEEG

**DOI:** 10.1002/epd2.70006

**Published:** 2025-03-04

**Authors:** Aayesha J. Soni, Ana Suller Marti, Giovanni Pellegrino

**Affiliations:** ^1^ Department of Clinical Neurological Sciences, Schulich School of Medicine and Dentistry Western University London Ontario Canada; ^2^ Department of Pediatrics, Schulich School of Medicine and Dentistry Western University London Ontario Canada

A 39‐year‐old gentleman was investigated with stereo‐electroencephalography (SEEG) in the context of multiple cavernomas (left posterior frontal and parietal) and recurrent seizures despite a previous left amygdalectomy. Abundant high‐amplitude activity in long runs was suspected to be epileptiform due to its morphology and close association with the left parietal cavernoma. With right hand movement and sleep, the discharges consistently attenuated, in contrast to other epileptiform discharges, leading us to conclude that it was mu rhythm (Figures 1 and 2).

**FIGURE 1:**
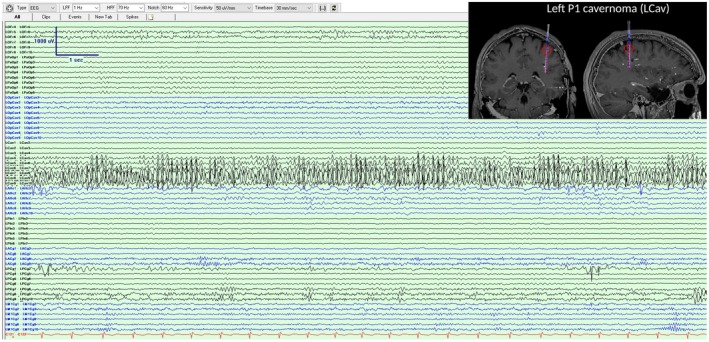
SEEG recording demonstrating abundant sharply contoured 13 Hz activity over LCav 7–10. Inset of coronal and sagittal MRI brain showing the left parietal cavernoma and the position of the SEEG electrode close to it, with affected contacts circled in red.

**FIGURE 2:**
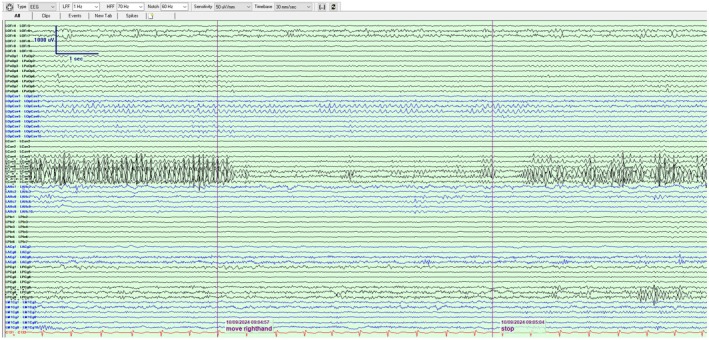
Previously described activity demonstrating attenuation with right‐hand movements, reappearing once it was stopped.

Mu rhythm, a well‐recognized variant of the dominant alpha/beta rhythms seen in the central sensori‐motor regions,^1^ is blocked by contralateral voluntary/passive movement or somatosensory stimulation.^2^ Its generating sources are located at the somatosensory cortex.^3^, ^4^, ^5^

To the best of our knowledge, there is only one other study that assessed mu in three patients with SEEG.^6^ Literature on the appearance of normal variants in SEEG is lacking. Care should be taken that physiological rhythms are not mistaken for epileptiform activity. When in doubt, reactivity and activity protocols can be helpful.

## FUNDING INFORMATION

No funding information.

## CONFLICT OF INTEREST STATEMENT

AJS has no relevant disclosures. ASM has no relevant disclosures. GP has no relevant disclosures.

## PATIENT CONSENT

Telephonic consent for publication was obtained from the patient.

**Test Yourself**

1. How can normal variant mu rhythm present on SEEG?
  - Rhythmic spike waves at low frequency.
  - Low voltage fast activity.
  - Apiculate/arciform alpha/beta (typically around 12 Hz in frequency) wave forms seen during normal wakefulness.
  - Mu rhythm is not seen on SEEG.
2. Where is mu rhythm most commonly seen?
  - Occipital cortex.
  - Sensori‐motor regions.
  - Superior temporal gyrus.
  - Premotor region.
3. What attenuates mu rhythm, both on scalp and stereo‐EEG?
  - Contralateral voluntary movement.
  - Contralateral somatosensory stimulation.
  - Normal sleep.
  - A + B + C.
  - Placing a white sheet of paper in front of the patient's eyes.

*Answers may be found in the* *Supporting information*.

## Supporting information


Data S1.

Data S2.

## Data Availability

The data that support the findings of this study are available from the corresponding author upon reasonable request.
