# Supplementary material for: Suspicious looking mu rhythm on SEEG
Source: Epileptic Disord. 2025 Mar 4;27(3):492–4. doi: 10.1002/epd2.70006 (PMC12203299; doi:10.1002/epd2.70006)
Supplement: Supplementary file 1 — Data S1. [file EPD2-27-492-s002.docx]

Multiple Choice Questions and Answers

1. How can normal variant mu rhythm present on SEEG?

A. Rhythmic spike waves at low frequency.

B. Low voltage fast activity.

C. Apiculate/arciform alpha/beta (typically around 12 Hz in frequency) wave forms seen during normal wakefulness.

D. Mu rhythm is not seen on SEEG.

(Answer: C)

2. Where is mu rhythm most commonly seen?

A. Occipital cortex.

B. Sensori-motor regions.

C. Superior temporal gyrus.

D. Pre-motor region.

(Answer: B)

3. What attenuates mu rhythm, both on scalp and stereo-EEG?

A. Contralateral voluntary movement.

B. Contralateral somatosensory stimulation.

C. Normal sleep.

D. A+B+C.

E. Placing a white sheet of paper in front of the patient's eyes.

(Answer: D)
