# Supplementary material for: Suspicious looking mu rhythm on SEEG
Source: Epileptic Disord. 2025 Mar 4;27(3):492–4. doi: 10.1002/epd2.70006 (PMC12203299; doi:10.1002/epd2.70006)
Supplement: Supplementary file 2 — Data S2. [file EPD2-27-492-s001.pptx]

## Slide 1
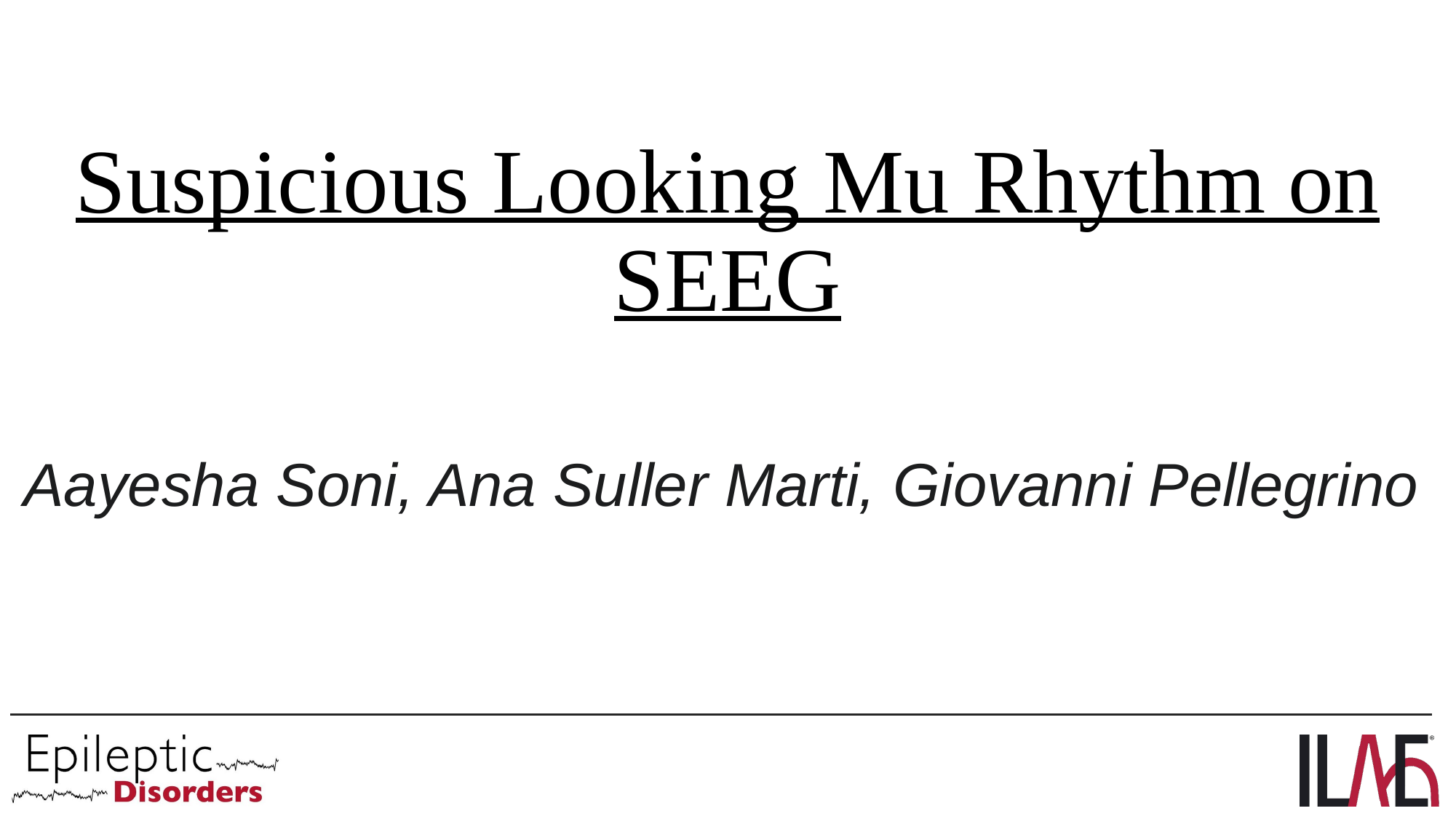

# Suspicious Looking Mu Rhythm on SEEG
Aayesha Soni, Ana Suller Marti, Giovanni Pellegrino

## Slide 2
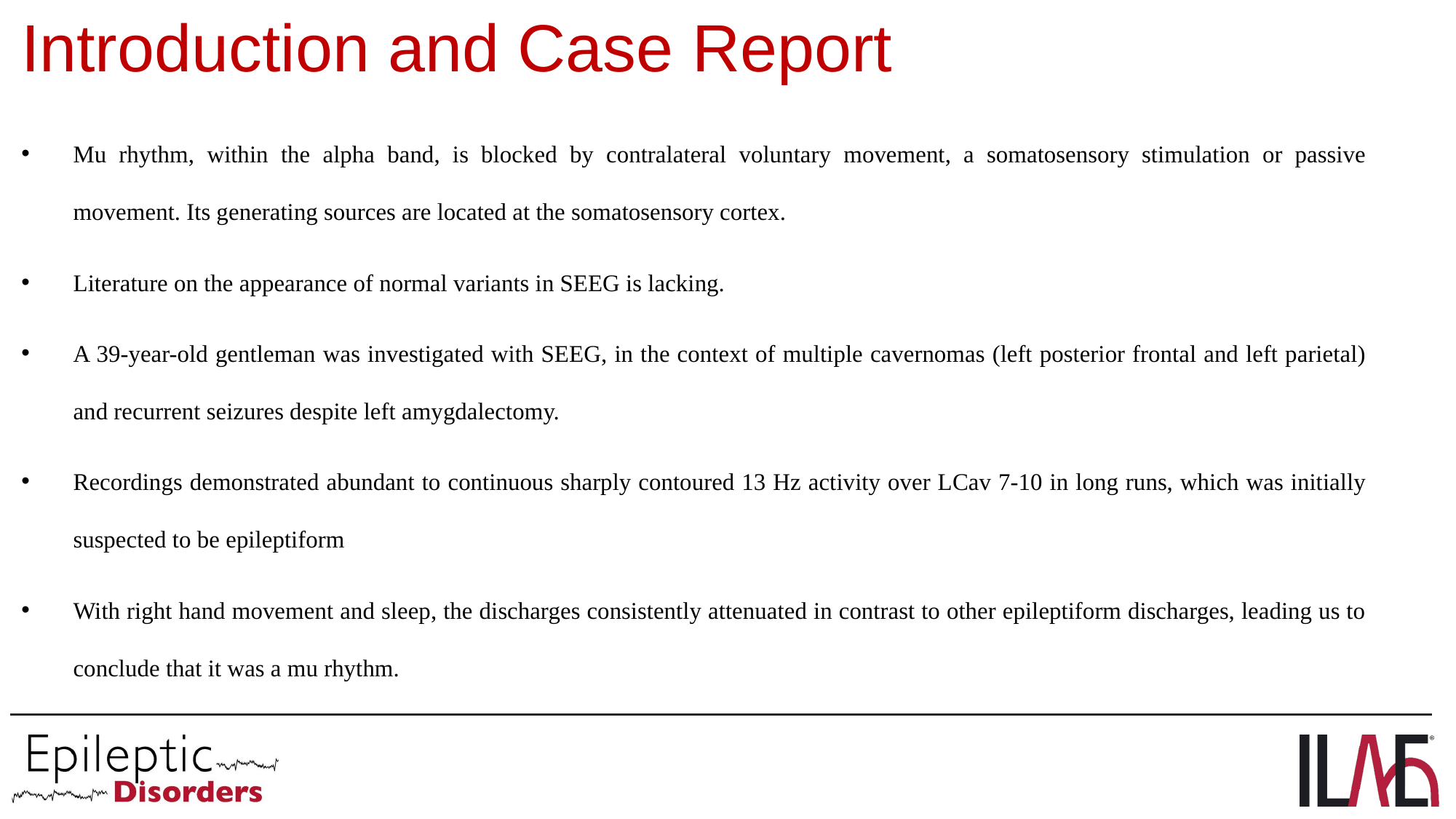

Introduction and Case Report
Mu rhythm, within the alpha band, is blocked by contralateral voluntary movement, a somatosensory stimulation or passive movement. Its generating sources are located at the somatosensory cortex.
Literature on the appearance of normal variants in SEEG is lacking.
A 39-year-old gentleman was investigated with SEEG, in the context of multiple cavernomas (left posterior frontal and left parietal) and recurrent seizures despite left amygdalectomy.
Recordings demonstrated abundant to continuous sharply contoured 13 Hz activity over LCav 7-10 in long runs, which was initially suspected to be epileptiform
With right hand movement and sleep, the discharges consistently attenuated in contrast to other epileptiform discharges, leading us to conclude that it was a mu rhythm.

## Slide 3
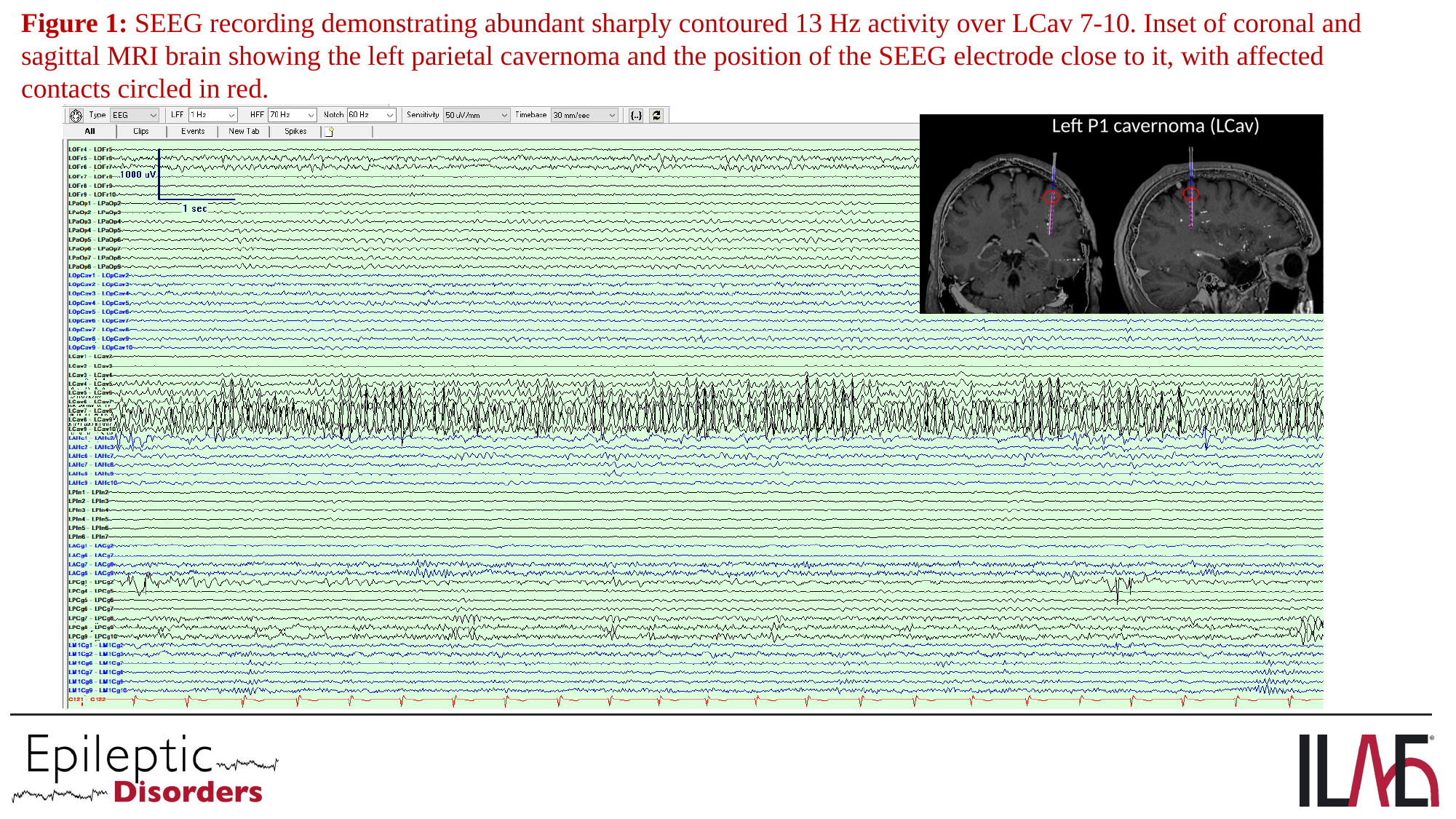

Figure 1: SEEG recording demonstrating abundant sharply contoured 13 Hz activity over LCav 7-10. Inset of coronal and sagittal MRI brain showing the left parietal cavernoma and the position of the SEEG electrode close to it, with affected contacts circled in red.

## Slide 4
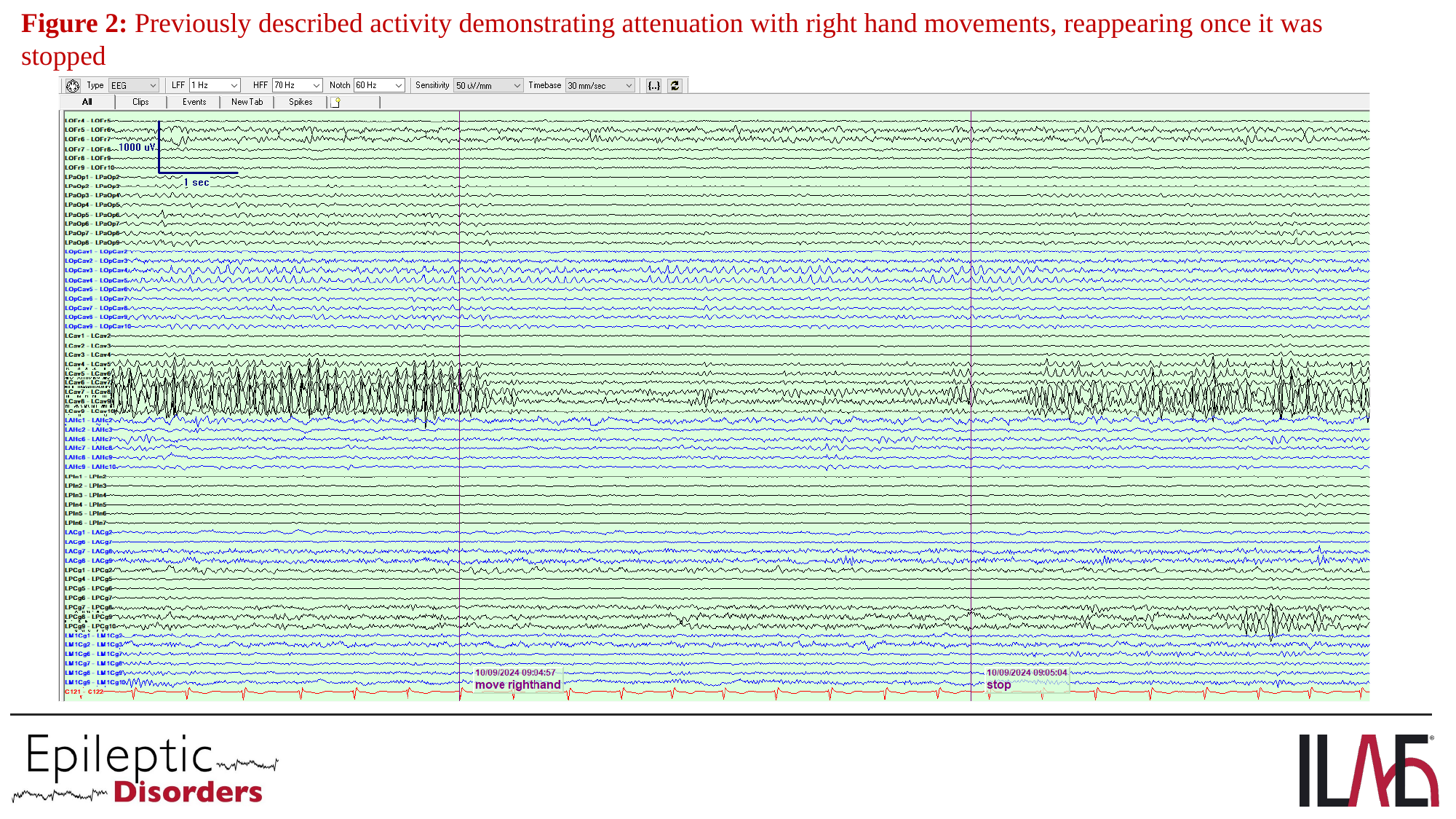

Figure 2: Previously described activity demonstrating attenuation with right hand movements, reappearing once it was stopped

## Slide 5
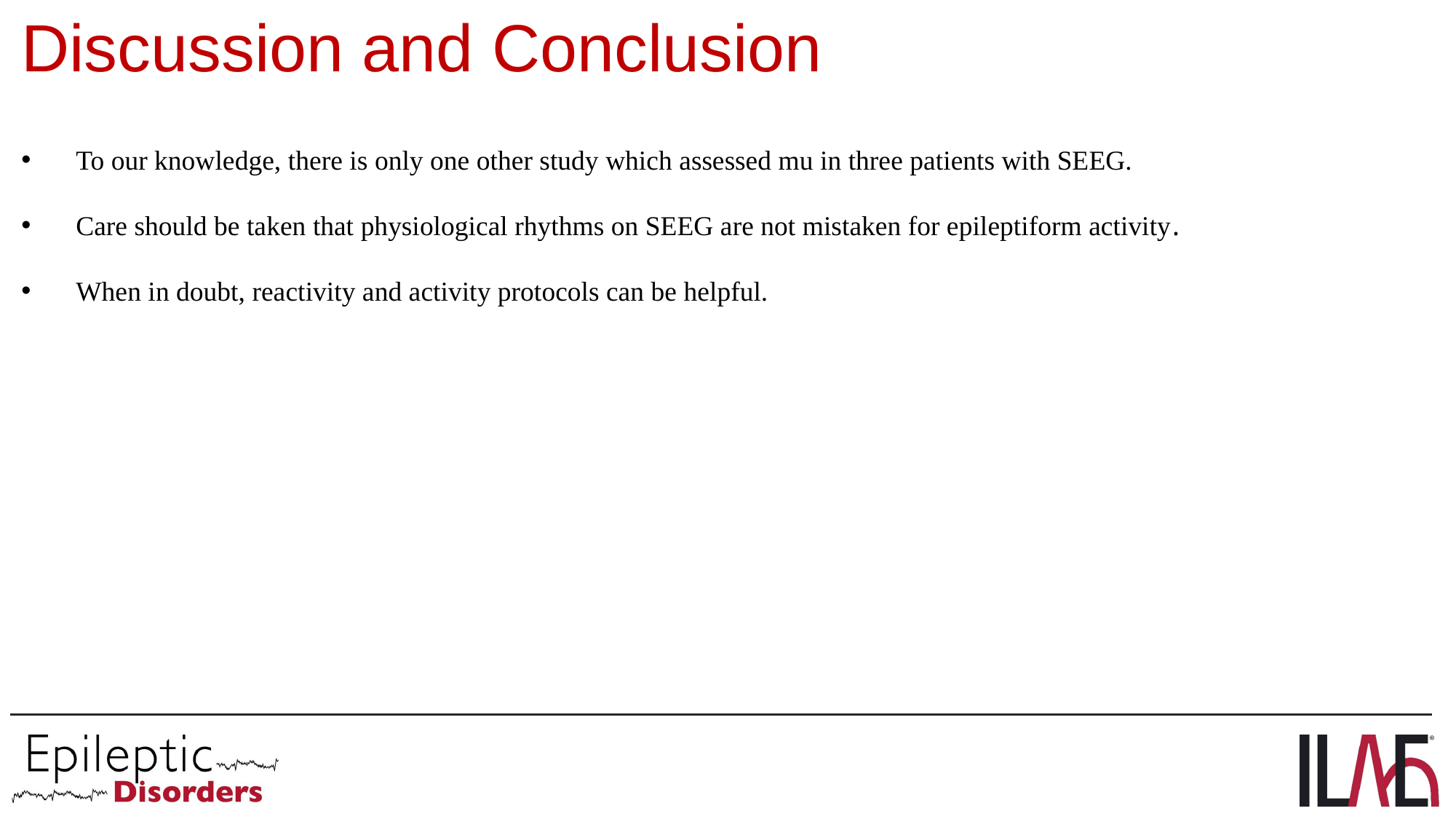

Discussion and Conclusion
To our knowledge, there is only one other study which assessed mu in three patients with SEEG.
Care should be taken that physiological rhythms on SEEG are not mistaken for epileptiform activity.
When in doubt, reactivity and activity protocols can be helpful.
